# Effects of Diets Combining Peanut Vine and Whole-Plant Corn Silage on Growth Performance, Meat Quality and Rumen Microbiota of Simmental Crossbred Cattle

**DOI:** 10.3390/foods12203786

**Published:** 2023-10-15

**Authors:** Jixiang Ma, Hua Liu, Mengqi Liu, Junying Xu, Jiading Lu, Shixi Cao, Shouren Li, Sen Ma, Zhichang Wang, Xiaoyan Zhu, Defeng Li, Hao Sun, Yinghua Shi, Yalei Cui

**Affiliations:** 1 College of Animal Science and Technology, Henan Agricultural University, Zhengzhou 450002, Chinayaleicui423@henau.edu.cn (Y.C.); 2 Henan Key Laboratory of Innovation and Utilization of Grassland Resources, Zhengzhou 450002, China; 3 Henan Forage Engineering Technology Research Center, Zhengzhou 450002, China

**Keywords:** antioxidant capacity, rumen fermentation, roughage, beef cattle

## Abstract

Peanut vine is a typical peanut by-product and can be used as a quality roughage resource. Whole-plant corn silage is a commonly used roughage. However, few studies have investigated the effects of diets combining peanut vine and whole-plant corn silage on growth performance, antioxidant capacity, meat quality, rumen fermentation and microbiota of beef cattle. To investigate these effects, eighty Simmental crossbred cattle (body weight, 451.27 ± 10.38 kg) approximately 14 months old were randomly divided into four treatments for a 90-day feeding experiment. A one-way design method was used in this experiment. According to the roughage composition, the cattle were divided into a control treatment of 45% wheat straw and 55% whole-plant corn silage (WG), and three treatments of 25% peanut vine and 75% whole-plant corn silage (LPG), 45% peanut vine and 55% whole-plant corn silage (MPG), and 65% peanut vine and 35% whole-plant corn silage (HPG), and the concentrate was the same for all four treatment diets. The results showed that compared to the WG group, the MPG group experienced an increase in their average daily feed intake of 14%, an average daily gain of 32%, and an increase in SOD activity in the spleen of 33%; in the meat, dry matter content increased by 11%, crude protein by 9%, and ether extract content by 40%; in the rumen, the NH_3_-N content was reduced by 36%, the relative abundance of *Firmicutes* increased, and the relative abundance of *Bacteroidetes* decreased (*p* < 0.05). These results showed the composition of 45% peanut vine and 55% whole-plant corn silage in the roughage improved growth performance, antioxidant capacity, meat quality, rumen fermentation, and microbiota of beef cattle.

## 1. Introduction

Beef, which is rich in amino acids and various trace elements, is highly sought after by consumers [1]. Currently, the massive consumption of beef is second only to that of pork and poultry, and as the quality of life of consumers is improving, the demand for high-quality beef is increasing [2]. According to data, the worldwide demand for beef was 70 million tons in 2019; it is estimated that the global demand for beef in 2023 will be approximately 74 million tons, with an the increase of approximately 4 million tons over the four years. The beef cattle industry is playing a crucial role in the global livestock farming industry [3]. However, the development of the beef cattle industry still needs to be improved regarding industry efficiency, meat quality, and ecological sustainability [4,5]. Improving the nutrition of livestock feed through an appropriate mix of feeds during the production and breeding process can be a cost-effective way to improve the production performance of beef cattle and meat quality [6].

Roughage is a crucial component of ruminant feeds, and its high crude fiber content is degraded and fermented in the rumen by rumen microbiota and then utilized by the body [7,8]. Peanut vine is a typical by-product of peanuts, and it can be used as a quality roughage resource [9]. It is estimated that the yield of peanut vine after peanut harvest accounts for about 60% to 65% of the total peanut production [10]. The crude protein (CP) content of peanut vine is about 12%, it is rich in vitamins and minerals [11], and its neutral detergent fiber (NDF) and acid detergent fiber (ADF) are about 50% and 36%, and are more easily digestible than alfalfa [12]. Quality CP in the diet is easy to digest and degrade, which promotes the synthesis of rumen microbial protein and the absorption of nutrients by the body [13]. Zhu et al. found that supplementing peanut vine in roughage for beef cattle compared to wheat straw could increase the CP content and linolenic acid level of longissimus dorsi muscle [14]. Abdou et al. found that the supplementation of peanut vine in millet stover diets for ruminants improved production performance and effectively reduced feeding costs [15]. Corn silage is an important source of fiber and energy for ruminants [16], with the protein content of about 8.8%, NDF content of about 50.3%, and ADF content of about 26.0% [17]. Zaralis et al. found that using corn silage as the sole forage in bull diets enhanced live weight gain [18]. A study has shown that supplementing corn silage to cattle diets compared to grass silage can increase dry matter (DM) intake and slightly increase carcass gain [19]. Phipps et al. found that supplementing corn silage in cow diets compared to first-cut perennial ryegrass silage increased forage DM intake and milk yield [20]. Therefore, supplementing ruminant diets with peanut vine or corn silage can increase the productivity of ruminants. It has been found that interactive effects exist in feeds for animals. A good combination of feeds would create more positive effects, which in turn would improve the efficiency of feed utilization [21].

We assumed that the diet combining 45% peanut vine and 55% whole-plant corn silage in roughage would enhance growth performance and meat quality of Simmental crossbred cattle. Accordingly, this research investigates the effects of the diet combining peanut vine and whole-plant corn silage on the growth performance, meat quality, and rumen microbiota of Simmental crossbred cattle.

## 2. Materials and Methods

### 2.1. Animals and Experimental Design

The study was conducted at Hengdu Xianan Cattle Farm (Henan, China), and all procedures followed the guidelines of the Animal Ethics Committee of Henan Agricultural University (approval HENAU-2022-015). To investigate the effects of diets combining peanut vine and whole-plant corn silage on beef cattle, eighty healthy Simmental crossbred cattle (body weight, 451.27 ± 10.38 kg) approximately 14 months old were selected for the experiment. A one-way design method was used in this experiment. The cattle were randomly divided into four treatments and tagged. Each treatment had four replicates, and each replicate contained five cattle. We ensured that the concentrate was the same in the diets for all four treatments, and the proportion of forage to concentrate was 44.09:55.91. The control treatment was the WG group (with roughage composition of 45% wheat straw and 55% whole-plant corn silage), and the three treatments were the LPG group (with roughage composition of 25% peanut vine and 75% whole-plant corn silage), the MPG group (with roughage composition of 45% peanut vine and 55% whole-plant corn silage), and the HPG group (with roughage composition of 65% peanut vine and 35% whole-plant corn silage). The experiment was conducted in a house with individual stalls and followed deworming and relative preventive measures. Five cattle were kept in an individual pen. The pre-test period was 7 d, and the formal period was 90 d. The diet composition and nutritional levels of all treatment groups are shown in Table 1, and the diets were prepared as a total mixed ration (TMR). The nutritional levels of the diets and the overall net energy were based on the Chinese Beef Cattle Feeding Standard [22]. The TMR chemical composition was determined according to Cavallini et al. [23]. The diet provided in this study was carefully monitored to ensure that aflatoxin levels were well below the established safety limits for animal feed. This precautionary measure was taken to safeguard the animals’ health and welfare. Aflatoxin contamination in animal feed can pose serious health risks, including impaired growth and liver damage. By maintaining feed quality within safe limits, we aimed to minimize any potential influence of aflatoxins on the study results [24]. The cattle were fed twice per day at 07:00 and 16:00, watered freely, and troughs and pens were cleaned regularly. When the experiment ended, one cow was randomly selected from the 5 cattle per replicate of each group, and a total of 16 cattle were selected for slaughter.

### 2.2. Sample Collection and Measurements

During the experiment, we collected and counted the amount of feed offered to and refused by each cow on a daily basis in the individual pen to calculate the average daily feed intake (ADFI). Individual body weight was measured on days 8 and 98 to calculate the average daily gain (ADG) and the feed/gain ratio (F/G). On the final morning of the experiment, before feeding, we collected blood samples (approximately 20 mL) from the tail vein of the 16 cattle selected for slaughter, collected them in a blood collection tube containing a procoagulant, rested the blood to clot, then separated the serum after 10 min at 3000 r/min at 4 °C and stored it at −20 °C. After 2 h of morning feeding, rumen fluid samples (200 mL) from the cattle selected for slaughter were collected using a rumen cannula and the pH was immediately measured using a PHS-3C acidity meter (Leijun Experimental Instrument Co., Ltd., Shanghai, China). Then the rumen fluid was filtered using four layers of gauze, and the rumen filtrate was used to determine rumen fermentation parameters such as ammonia nitrogen (NH_3_-N) and volatile fatty acids (VFA) using the method described by Du et al. [25].

According to the requirements of the slaughtering experiment, the experiment cattle had fasted for 24 h and were prevented from drinking water for 20 h before slaughter. The slaughtering process was conducted by Henan Hengdu Food Co., Ltd. (Zhumadian, China), using hanging slaughtering methods and strictly followed animal welfare and food hygiene regulations. After slaughtering, the heart, liver, spleen, lung, and kidney were obtained from the abdominal cavity for organ index calculation; organ index = (organ weight/body weight). Samples were taken from the same part of the liver and spleen and placed in 5 mL cryovials, which were then quickly frozen and stored in liquid nitrogen.

Carcass weight was determined by weighing the carcasses after cooling at 0–2 °C for 48 h. Dressing percentage = (carcass weight/body weight) × 100%, the meat and bones were separated and weighed separately to calculate the net meat weight, the meat/bone ratio (meat weight/bone weight), and carcass meat yield (net meat weight/carcass weight). The 12–13th rib of the left half carcass was cut, and then a grid-like sulfuric acid paper was used on the cut surface to trace the outline of the eye muscle along the muscle edge, and the eye muscle area was calculated. The longest part of the dorsal muscle between the 12th and 13th rib of the left half of the carcass was divided into two parts. One part was used to measure the physical properties of the muscle (marbling score, shear force, cooking yield, water holding capacity, and pH), and the other part was used to measure the nutritional components (DM, CP, crude fat, and crude ash) and fatty acid composition of the muscle, as described by Zhu et al. [14].

### 2.3. Determination of Antioxidant Indicators

Superoxide dismutase (SOD), glutathione peroxidase (GSH-Px), malondialdehyde (MDA) content, and total antioxidant capacity (T-AOC) were determined using the kits (Nanjing Jiancheng Bioengineering Institute, Nanjing, China) [26]. Measurements were repeated multiple times to ensure accuracy.

### 2.4. Detection of Liver Lipid Metabolism Genes

The extraction of liver tissue RNA was performed according to the instructions of the RNAeasy kit. RNA concentration was detected using NanoDrop 2000 UV-vis spectrophotometer (Thermo Scientific, Wilmington, NC, USA). cDNA was synthesized using a reverse transcription kit (Vazyme, Nanjing, China) [27]. Gene expression levels were detected by Lightcycle 96 real-time PCR instrument (Roche, Basel, Switzerland) using the real-time fluorescence quantification kit (Vazyme, Nanjing). β-actin was used as the internal reference gene, and the 2^−△△Ct^ method was used to calculate the expression levels of genes. Quantitative primers were designed by the NCBI online website (National Center for Biotechnology Information (nih.gov), accessed on 2 March 2023). Table S1 shows the primers synthesized by the Shangya Biotechnology Co., Ltd. (Zhengzhou, China).

### 2.5. 16s rRNA Sequencing and Bioinformatics Analysis

Microbial community genomic DNA was extracted from rumen fluid samples using the E.Z.N.A.^®^ soil DNA Kit (Omega Bio-tek, Norcross, GA, USA). The V3-V4 region of the bacterial 16S rRNA was amplified using the 338F (5′-ACTCCTACGGGAGGCAGCAG-3′) and 806R (5′-GGACTACHVGGGTWTCTAAT-3′) primers [28]. A TruSeqIM DNA sample prep kit was used to build the library and sequenced by Illumina’s MiSeq PE300 platform. The Shannon and Chao indexes of α-diversity and principal coordinate analysis (PCoA) were conducted using the Majorbio Cloud Platform (www.majorbio.com, accessed on 26 March 2023). To determine differences between the groups, the Kruskal Wallis H test was used. The Spearman correlation analysis was performed to determine the effect of microbiota at the genus level interacting with apparent performance and the predicted functional pathways. PICRUSt was used to explore differences in the KEGG pathway between all groups to predict the correlative microbial functional pathways involved. The raw reads were deposited into the NCBI database (BioProject: PRJNA978324).

### 2.6. Statistical Analysis

All data were tested for normality and homogeneity before analysis. Data were analyzed using one-way analysis of variance (ANOVA) through SPSS 20.0, and differences between means were analyzed using Duncan’s test. The *p*-value of <0.05 was defined as the significance criterion for the difference. Data were presented as mean ± standard deviation.

## 3. Results

### 3.1. Growth Performance 

As shown in Table 2, the treatment groups had no significant differences in initial weight and F/G. The ADG of the LPG and MPG groups was significantly higher than that of the WG group (*p* < 0.05), and the ADG was approximately 32% higher in the MPG group compared to the WG group. The final weight of the MPG group was significantly higher than that of the WG group (*p* < 0.05). Additionally, the ADFI of the HPG and MPG groups was significantly higher than that of the WG group (*p* < 0.05), and the ADFI was approximately 14% higher in the MPG compared to the WG group.

### 3.2. Organ Index

We found that diets combining peanut vine and whole-plant corn silage did not affect the organ index of Simmental crossbred cattle (Table S2).

### 3.3. Serum and Tissue Antioxidant Capacity

**Figure 1:**
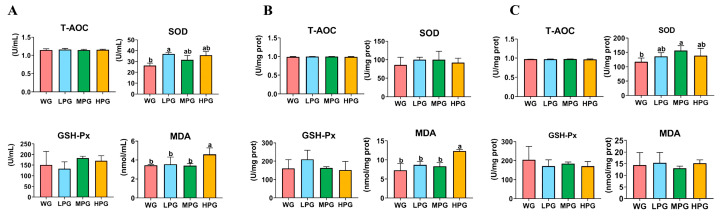
The effects of diets combining peanut vine and whole-plant corn silage on the antioxidant capacity of Simmental crossbred cattle serum (**A**), liver (**B**), and spleen (**C**). Different letters in the same row indicate significant differences (*p* < 0.05).

In serum, the MDA activity of the HPG group and the SOD activity of the LPG group were significantly higher than those of the WG group (*p* < 0.05) (Figure 1A). Nonetheless, in the liver, the MDA activity of the HPG group was significantly higher than that of the WG group (*p* < 0.05) (Figure 1B). Furthermore, in the spleen, the SOD activity was approximately 33% higher in the MPG compared to the WG group (*p* < 0.05) (Figure 1C).

### 3.4. Slaughter Performance and Muscle Physical Properties

As shown in Table 3, the MPG group had significantly increased carcass weight and net meat weight compared to the HPG group (*p* < 0.05). The MPG group had a significantly increased eye muscle area compared to the LPG group (*p* < 0.05). Compared to the WG group, the MPG group had an increased eye muscle area (*p* = 0.059). However, the treatment groups had no significant differences regarding dressing percentage, carcass meat yield, meat/bone ratio, marbling score, cooking yield, water holding capacity, shear force, pH45min, and pH48.

### 3.5. Conventional Nutritional Composition and Fatty Acid Content of the Muscle 

As shown in Table 4, the EE content in the muscle of the LPG group was significantly higher than that in the WG group and HPG group (*p* < 0.05). The DM, CP, and EE content in the muscle of the MPG group were significantly higher than those in the WG group (*p* < 0.05), and the CP content in the muscle of the MPG group was significantly higher than that in the LPG group (*p* < 0.05). The EE content in the muscle of the HPG group was significantly higher than that in the WG group (*p* < 0.05). There was no significant difference in the ash content among the treatment groups. The linoleic acid content in the LPG and HPG groups was significantly higher than that in the WG group (*p* < 0.05) (Table 5). 

### 3.6. The Expression of Liver Lipid Metabolism Genes

**Figure 2:**
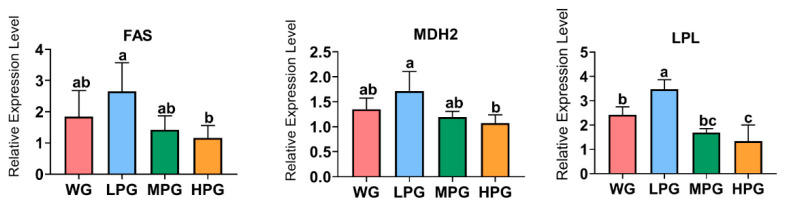
The effects of diets combining peanut vine and whole-plant corn silage on the expression of liver lipid metabolism genes in Simmental crossbred cattle. *FAS*, fatty acid synthase; *MDH2*, malate dehydrogenase 2; *LPL*, lipoprotein lipase. Different letters in the same row indicate significant differences (*p* < 0.05).

As shown in Figure 2, the relative expression levels of *FAS* and *MDH2* genes in the liver of the LPG group were significantly higher than those in the HPG group (*p* < 0.05). Additionally, the expression level of the *LPL* gene in the liver was significantly higher in the LPG group than in the other three groups (*p* < 0.05).

### 3.7. Rumen Fermentation Parameters

The NH_3_-N content in the rumen fluid of the MPG and HPG groups was significantly lower than that in the WG and LPG groups (*p* < 0.05). The acetate and propionate contents in the LPG group were significantly higher than those in the MPG group (*p* < 0.05). The A/P ratio in the HPG group was significantly higher than that in the WG, LPG, and MPG groups (*p* < 0.05) (Table 6).

### 3.8. Rumen Microbial Communities

**Figure 3:**
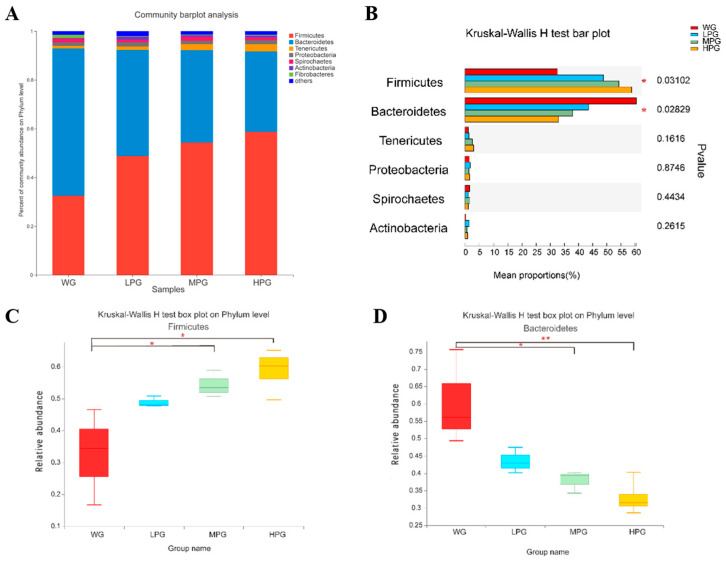
(**A**) The effects of diets combining peanut vine and whole-plant corn silage on the rumen microbial community composition of Simmental crossbred cattle at the phylum level. (**B**) Differential microbiota in the rumen of Simmental crossbred cattle at the phylum level. (**C**) Analysis on the difference of *Firmicutes* abundance. (**D**) Analysis on the difference of *Bacteroidetes* abundance. * *p* < 0.05, ** *p* < 0.01.

**Figure 4:**
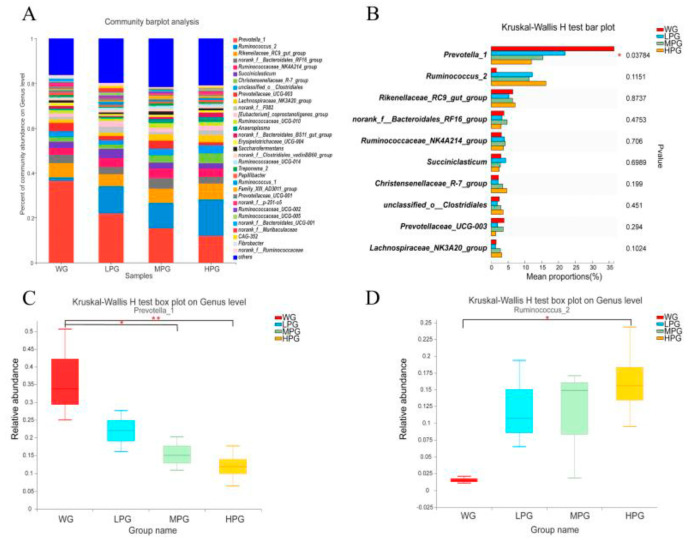
(**A**) The effects of diets combining peanut vine and whole-plant corn silage on the rumen microbial community composition of Simmental crossbred cattle at the genus level. (**B**) Differential microbiota in the rumen of Simmental crossbred cattle at the genus level. (**C**) Analysis on the difference of *Prevotella_1* abundance. (**D**) Analysis on the difference of *Ruminococcus_2* abundance. * *p* < 0.05, ** *p* < 0.01.

Firstly, the microbiota of rumen fluid samples was deeply sequenced (Figure S1). Analysis of the microbial diversity and richness of the rumen microbiota in the WG, LPG, MPG, and HPG groups revealed no significant differences in the Shannon index (Figure S2A); however, the Chao index in the HPG group was significantly higher than in the WG group (*p* < 0.05) (Figure S2B). The results of PCoA showed that the rumen microbiota composition and structure in the WG group differed from that in the LPG, MPG, and HPG groups, while there was some similarity in the rumen microbiota composition in the LPG, MPG, and HPG groups (Figure S2C). At the phylum level, *Firmicutes*, *Bacteroidetes*, and *Tenericutes* were the dominant bacterial phyla in all four treatment groups and accounted for more than 90% of the total bacterial phyla. *Firmicutes* accounted for 32.53%, 48.81%, 54.29%, and 58.75% of the total bacterial phyla in the WG, LPG, MPG, and HPG groups, respectively. *Bacteroidetes* accounted for 60.37%, 43.48%, 37.94%, and 32.96% of the total bacterial phyla, respectively, while *Tenericutes* accounted for 1.06%, 1.38%, 2.46%, and 2.95% (Figure 3A). There were significant differences in *Firmicutes* and *Bacteroidetes* among the four treatment groups (Figure 3B). Compared to the WG group, the relative abundance of *Firmicutes* was significantly increased in the MPG and HPG groups (*p* < 0.05) (Figure 3C). Compared to the WG group, the relative abundance of *Bacteroidetes* was significantly decreased in the MPG and HPG groups (*p* < 0.05) (Figure 3D). At the genus level, *Prevotella_1*, *Ruminococcus_2*, and *Rikenellaceae_RC9_gut_group* were the dominant genera in all four treatment groups (Figure 4A). *Prevotella_1* was significantly different among the four treatment groups (Figure 4B). Compared to the WG group, the relative abundance of *Prevotella_1* significantly decreased in the MPG and HPG groups (*p* < 0.05) (Figure 4C). The relative abundance of *Ruminococcus_2* was significantly increased in the HPG group compared to the WG group (*p* < 0.05) (Figure 4D).

### 3.9. Functional Prediction of Rumen Microbiota and Correlation Analysis

**Figure 5:**
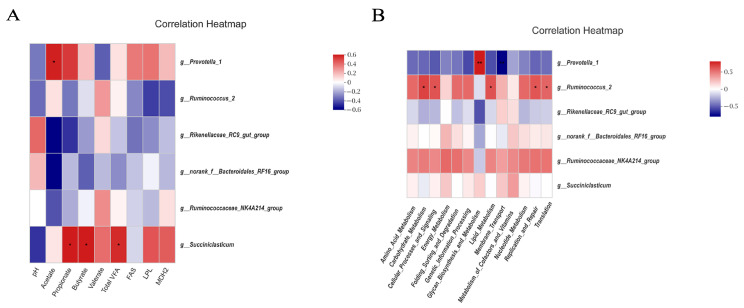
(**A**) Correlation analysis between rumen microbes and environmental factors. (**B**) Correlation analysis of rumen microbes and the predicted functional pathways. Total VFA, total volatile fatty acid; *FAS*, Fatty acid synthase; *MDH2*, Malate dehydrogenase 2; *LPL*, Lipoprotein lipase. * *p* < 0.05, ** *p* < 0.01.

To further explore the correlations between rumen microbiota at the genus level, metabolites, and lipid metabolism genes, Spearman correlation analysis was conducted. The results showed that *Prevotella_1* was positively correlated with acetate content (r > 0.5, *p* < 0.05), and *Succiniciasticum* was positively correlated with propionate, butyrate, and the total VFA content (r > 0.5, *p* < 0.05) (Figure 5A). A further prediction of the KEGG metabolic function of rumen microbiota was carried out using PICRUSt, and the results showed that the KEGG metabolic function of rumen microbiota in the four treatment groups changed. PICRUSt analysis showed that the different ratios of peanut vine to whole-plant corn silage significantly impacted rumen microbiota metabolic pathways in cattle. Compared to the WG group, the functional genes related to membrane transport, lipid metabolism, and transcription were up-regulated in the LPG, MPG, and HPG groups (*p* < 0.05). In contrast, genes related to folding, sorting and degradation, and glycan biosynthesis and metabolism were down-regulated (*p* < 0.05) (Figure S3). Correlation analysis of rumen microbiota and the predicted functional pathways showed that *Prevotella_1* was positively correlated with glycan biosynthesis and metabolism (r > 0.5, *p* < 0.01) and negatively correlated with membrane transport (r < −0.5, *p* < 0.01). *Ruminococcus_2* was positively correlated with carbohydrate metabolism, cellular processes and signaling, lipid metabolism, replication and repair, and translation (r > 0.5, *p* < 0.05) (Figure 5B).

## 4. Discussion

The study aimed to investigate the effects of diets combining peanut vine and whole-plant corn silage on beef cattle. Roughage is an extremely important component of ruminant feeds, and high-quality roughage can improve the efficiency of ruminant farming, as well as the composition of it making a significant influence on the growth performance of cattle [29]. We found that diets combining peanut vine and whole-plant corn silage improved the growth performance of beef cattle, especially the ADG of the MPG group which was the highest; this result was similar to Abdou et al. [15]. This is probably due to the lower nutritional value of the roughage in the WG group, such as the much lower CP content compared to the LPG, MPG and HPG groups [30], making it difficult to provide sufficient nutrients for ruminants. Moreover, the ratio of peanut vine to whole-plant corn silage in the MPG group can have a greater positive combination effect and improve the production performance of the cattle [21]. On the other hand, the growth performance of the MPG group was higher than that of the WG group, which may be due to the higher fiber and lignin content of wheat straw compared to peanut vine, which can decrease the degradation of it by rumen microbiota, thus reducing feed digestion and utilization by the body. However, the organ indexes of the treatment groups indicated that the supplementation of peanut vine in the roughage of cattle would not affect the growth and development of organs.

Antioxidation is an active and spontaneous self-protection mechanism of the animal body, and antioxidant enzymes can enhance the immunity and defense of the body and reduce the damage caused by free radicals [31]. The activities of T-AOC, GSH-Px, SOD, and MDA can be used to reflect the antioxidant capacity of the animal. T-AOC is a comprehensive indicator of antioxidant capacity [32]. GSH-Px and SOD are two important antioxidant enzymes whose main role is eliminating free radicals in the body [33]. MDA can damage the integrity of tissues and cells, which can lead to cell damage. In this study, the SOD activity in spleen of the MPG group was higher than that of the WG group. This result shows the diet combining 45% peanut vine and 55% whole-plant corn silage improved the antioxidant capacity of the body. It is probably due to some bioactive components such as flavonoids in peanut vine that improve the antioxidant capacity [34], and it could also be one of the reasons that the diet combining peanut vine and whole-plant corn silage improves the performance of the cattle.

Slaughter performance is an essential indicator in the beef industry. Our study found that the MPG group was higher than the other groups with regard to carcass weight, net meat weight, and eye muscle area, indicating that the diet combining 45% peanut vine and 55% whole-plant corn silage could improve the slaughter performance of cattle. It could be due to the increased ADFI and ADG of the beef cattle in the MPG group, which thus increased the digestion of the feed by the body, and improved nutrient deposition in the body. On the other hand, it could be due to the MPG group’s roughage combination producing more positive effects. 

Changes in EE and CP content in muscle can affect the sensory properties and nutritional traits of beef. In this study, we found that the DM, CP and EE content in the muscle of the MPG group was significantly higher than that of the WG group. The results indicate that the diet combining 45% peanut vine and 55% whole-plant corn silage produced a more positive effect, enhanced the absorption of nutrients from the feed by the body, and thus promoted the deposition of nutrients in the muscle. Interestingly, we found that the LPG group had the highest EE content in muscle of the treatment groups and that the supplementation of 25% peanut vine in the roughage might be the optimum ratio to promote muscle fat deposition in cattle. Linoleic acid, linolenic acid, and arachidonic acid as PUFA in the muscle have been found to reduce cardiovascular diseases, lower cholesterol, and reduce hypertension [35]. We found that the content of linoleic acid in the muscle of the LPG and HPG groups was higher than that of the WG group. This is probably because the PUFA content of wheat straw, which belongs to graminoid forage, is lower than that contained in peanut vine, resulting in differences in the linoleic acid content of beef among the treatment groups [14]. As an essential organ of the body, the liver is where most of the metabolic activities of the body, such as the oxidation of fatty acids and the synthesis of lipids such as triglycerides and cholesterol, take place. FAS, MDH, and LPL are crucial enzymes involved in lipid metabolism in the body. FAS is a crucial enzyme in the de novo synthesis of fatty acids, and its activity can be used to determine the capacity of fatty acid synthesis in the liver. MDH is involved in the tricarboxylic acid cycle in the body and plays essential roles in the synthesis of fatty acids. In contrast, LPL is a crucial enzyme in lipolysis metabolism, which is mainly secreted and expressed by adipocytes, myocytes, and macrophages [36]. We found that cattle in the LPG group increased the gene expression levels of *FAS* and *MDH2*. The results indicate that the supplementation of appropriate amounts of peanut vine can promote the synthesis of fatty acids, thus increasing the fat deposition in the liver, which can further increase triglycerides and cholesterol levels in the liver to promote the deposition of fat in the muscle. Interestingly, the beef cattle in the LPG group also increased the gene expression level of *LPL*, which in turn accelerated fat catabolism. From our results, it seems that the diet combining 25% peanut vine and 75% whole-plant corn silage would eventually promote fat deposition, probably due to the efficiency of lipid synthesis being higher than that of lipolysis in the body.

Ruminal pH reflects the state of rumen fermentation, which is around 6.6 in normal conditions. Mould et al. found that rumen degradation of cellulose was decreased when the pH in the rumen was below 6.0, thereby reducing the digestibility of the feed [37]. We found the rumen pH was in the normal range in all groups, indicating that feeding different ratios of peanut vine to whole-plant corn silage combinations cannot affect ruminal pH in the cattle. NH_3_-N is a degradation product of protein, amino acids, and non-protein nitrogen in the feed, reflecting the use of ammonia by rumen microbiota and the efficiency of the nitrogen degradation in the rumen. However, excessively high NH_3_-N concentration would cause nitrogen losses, and excessively low NH_3_-N concentration would reduce rumen degradation of fiber and the productivity of rumen microbial protein synthesis. In this study, NH_3_-N concentration ranged from 6.95 to 12.08 mg/dl, within the normal range for all groups. The NH_3_-N concentration in the MPG group was significantly lower than that in the WG group. However, the rate of protein degradation in the rumen was affected by the quality of CP in the diet. The result might be due to the fact that the quality of CP in the diet combining peanut vine and whole-plant corn silage was greater than that in the diet combining wheat straw and whole-plant corn silage, which accelerated the degradation of protein in the rumen, and facilitated the synthesis of bacterial protein by the rumen microbiota using NH_3_-N, thus providing energy for the growth of the body, which laterally explains the higher ADG in the MPG group. Ruminal VFA is produced by the fermentation of carbohydrates in the feed by rumen microbiota [38]. VFA can provide the body with energy and can also participate in various physiological processes such as the tricarboxylic acid cycle, the transformation and storage of glucose, and the formation of lipid, thus affecting overall health and performance. Raspa et al. found that a high-fiber diet significantly influenced VFA content in the horse gut, which is different from the results of the present study. This finding indicates that differences in animal species, fermentation sites, and feed sources can influence the VFA content in the body [39]. The A/P ratio can reflect the utilization of nutrients by the body of the animal. We found that the A/P ratio of all treatment groups was within the normal range in this study, and the A/P ratios of the LPG and MPG groups were lower than those of the HPG group, which were more beneficial to nutrient absorption and utilization. It is easy to see that the diet combining 45% peanut vine and 55% whole-plant corn silage improved rumen fermentation.

The rumen microbiota can degrade the majority of organic and fibrotic material in feed and plays essential roles in the nutritional metabolism, digestion, and absorption of ruminants. We found no significant differences in microbial α-diversity in all the treatments of diets combining peanut vine and whole-plant corn silage. At the phylum level, the dominant phylum of rumen microbiota were *Firmicutes*, *Bacteroidetes*, and *Tenericute* in all the treatments, and the relative abundances of *Firmicutes* and *Bacteroidetes* were higher. Chang et al. found that *Firmicutes* and *Bacteroidetes* were higher in relative abundance in the rumen of cattle [40], which is similar to this study. Furthermore, Myer et al. found *Firmicutes* and *Bacteroidetes* as the two dominant bacterial phyla in the rumen of ruminants [41]. It was found that the relative levels of *Firmicutes* and *Bacteroidetes* could be used to assess the energy requirements of ruminants [42]. *Firmicutes* can produce a diverse range of lipases, proteases, cellulases, etc., thereby hydrolyzing complex macromolecular compounds such as fats, cellulose, and glycans [43]. *Bacteroidetes* are generally regarded as having a role in degrading such proteins and carbohydrates [44]. It was reported that the relative abundance of *Firmicutes* was higher than that of *Bacteroidetes* in obese people and obese mice. The obese mice fecal bacteria transplantation to the germ-free mice increased in fat deposition, so *Firmicutes* may be related to lipid metabolism in the body [45]. We found the abundance of *Firmicutes* gradually increased, and the abundance of *Bacteroidetes* gradually decreased at the phylum level with increasing supplementation of peanut vine in the roughage. This suggests that diets combining peanut vine and whole-plant corn silage increased the abundance of *Firmicutes*, promoted the deposition of body fat, and improved the production performance of beef cattle.

In-depth analysis of the rumen microbiota found that the dominant genera were mainly *Prevotella_1* and *Ruminococcus_2* at the genus level in all the treatment groups. During the fermentation process in the rumen, *Prevotella_1* is one of the most commonly abundant genera. It acts as the main protein-degrading bacteria in the rumen to digest proteins, peptides, and starch-active substances. It cannot degrade fiber directly but can coexist with fiber-degrading bacteria to indirectly promote fiber degradation and plays essential roles in the metabolism in the rumen [46]. We found that the relative abundance of Prevotella_1 in the rumen of the treatments was lower than that of the WG group, but it was still the dominant bacteria in the rumen, similar to the results of Jami et al. [47]. This may be due to the higher content of degradable fiber in the diets combining peanut vine and whole-plant corn silage, which affects the rumen microbiota and increases the abundance of fiber-degrading bacteria, thereby affecting the relative abundance of the *Prevotella* [48]. *Ruminococcus* is a group of fiber-degrading bacteria in the rumen which plays a major role in fiber degradation and can degrade cellulose and hemicellulose in plant-based feed [49]. We found that the relative abundance of *Ruminococcus_2* in the rumen of the HPG group was significantly higher than that of the WG group, which is likely to be related to the increase in degradable fiber content in the feed. Pitta et al. found that *Fibrobacter* and *Ruminococcus* had a tendency to increase in the rumen of cattle with increased amounts of hay [50]. The results were similar to this study. The relative abundance of *Ruminococcus_2* was lower in the WG group. This may be due to the higher content of difficult to degrade fiber such as lignin in the diet combining wheat straw and whole-plant corn silage, which affects the colonization of fiber-degrading bacteria in the rumen, thus causing changes in the rumen microbiota. But the higher abundance of *Prevotella_1* in the rumen can be used to assist the degradation of fiber so that the body obtains sufficient nutrients. In addition, Ley et al. found that *Prevotella* was associated with inflammation in the body [51]. In this study, the growth performance of the WG group was poorer than that of the peanut vine treatment groups, probably due to the higher relative abundance of *Prevotella_1* in the WG group, which affected the health status of the body, which in turn affected the digestion and utilization of various nutrients by the rumen. The rumen is a crucial digestive organ for ruminants, with more than 70% of the feed consumed being degraded by the microbiota in the rumen and eventually digested and absorbed by the body. Saleem et al. argued that about 55–60% of rumen metabolites are related to rumen microbes [52], and the microbes are closely linked to muscle fat deposition in the body. Correlation analysis of rumen microbes with rumen metabolites and lipid metabolism genes showed that *Prevotella_1* was positively correlated with acetate level, which is probably because *Prevotella* can effectively degrade xylan, xyloglucan, and pectin and translates sugars into acetate, succinate, and propionate [53]. The main metabolic function of microbes that participate in the metabolic processes of the host by utilizing carbohydrates hardly utilized and absorbed by the host itself, plays a crucial role in rumen metabolism. Therefore, PICRUSt analysis was carried out based on the results of rumen microbial sequencing and found that functions such as lipid metabolism and membrane transport were up-regulated in the treatments that were fed the diets combining peanut vine and whole-plant corn silage. It is probably because *Ruminococcus* and other fiber-degrading bacteria can degrade fiber and produce acetate, propionate, and butyrate in the rumen. VFA can improve carbohydrate utilization, promoting lipid metabolism in the body. In correlation analysis of the predicted functional pathways and bacteria, we found that *Ruminococcus_2* was positively correlated with carbohydrate metabolism, while *Ruminococcus* belongs to the group of fiber-degrading bacteria that can hydrolyze and ferment carbohydrates which plays essential roles in rumen fermentation, further confirming our findings. Moreover, we found that *Prevotella_1* was positively correlated with glycan biosynthesis and metabolism, demonstrating that *Prevotella* plays essential roles in the digestion and utilization of glycans and plant polysaccharides. 

## 5. Conclusions

In conclusion, the diet combining 45% peanut vine and 55% whole-plant corn silage improved the performance of beef cattle. For growth performance, ADFI and ADG were increased by 14% and 32%, respectively; for antioxidant capacity, SOD activity in the spleen was increased by 33%; for meat quality, DM, CP, and EE in meat were increased by 11%, 9%, and 40%, respectively; for rumen fermentation, the NH_3_-N content was decreased by 36%; and for rumen microbiota, it increased relative abundance of *Firmicutes* and decreased relative abundance of *Bacteroidetes*. This study indicates that the diet could modulate the rumen microbiota, improve rumen fermentation and metabolic pathways, and improve meat quality and growth performance.

## 6. Future Implications

In this study, we found that a diet combining peanut vine and whole-plant corn silage improved the growth performance and meat quality of beef cattle. We believe that the diet in this study is more suitable for areas with high peanut production, which is not only effective in reducing beef production costs, but also utilizing by-products of peanuts in an environmentally friendly way. However, there are some limitations of this study, such as lack of consideration of forage palatability and the lack of in-depth mechanistic exploration of the results. In future studies, we would consider exploring the mechanisms and conducting research on forage palatability to provide more meaningful references for the beef cattle industry.

## Figures and Tables

**Table 1:** Diet composition and nutritional levels (dry matter basis).

Items	Treatments			
	WG	LPG	MPG	HPG
Ingredient (%)				
Whole-plant Corn Silage	24.26	33.04	24.26	15.43
Wheat Straw	19.83			
Peanut Vine		11.05	19.83	28.66
Concentrate ^1^	55.91	55.91	55.91	55.91
Total	100.00	100.00	100.00	100.00
Nutritive level ^2^				
NE_mf_, (MJ/kg)	6.13	6.60	6.63	6.66
CP	10.24	11.27	11.59	11.91
NDF	28.34	22.51	23.37	24.24
ADF	18.50	14.93	16.28	17.65

^1^ Ingredients of concentrate were 60% corn, 10% DDGS, 10% soybean meal, 13% wheat bran, 1% limestone, 2% NaHCO_3_, and 4% premix. The premix provided the following per kg of diets: Fe 1200 mg, Zn 450 mg, Cu 150 mg, Se 5 mg, I 15 mg, Co 4 mg, VA 150,000 IU, VD_3_ 50,000 IU, and VE 500 mg. ^2^ NE_mf_ was the calculated value, NE_mf_ = comprehensive net energy = maintain net energy (NEm) + net energy of weight gain (NE_g_).

**Table 2:** The effects of diets combining peanut vine and whole-plant corn silage on the growth performance of Simmental crossbred cattle.

Parameters of Interest	Treatments			
	WG	LPG	MPG	HPG
Initial weight, kg	455.67 ± 6.72	441.58 ± 10.14	449.83 ± 7.97	456.67 ± 14.25
Final weight, kg	547.00 ± 9.18 ^b^	552.25 ± 3.14 ^b^	570.50 ± 13.07 ^a^	565.83 ± 16.10 ^ab^
ADFI, kg/d	9.25 ± 0.19 ^c^	9.45 ± 0.10 ^c^	10.56 ± 0.10 ^a^	9.88 ± 0.15 ^b^
ADG, kg/d	1.04 ± 0.08 ^b^	1.26 ± 0.15 ^a^	1.37 ± 0.14 ^a^	1.24 ± 0.14 ^ab^
F/G	8.95 ± 0.58	7.60 ± 0.97	7.76 ± 0.81	8.04 ± 0.91

Different letters in the same row indicate significant differences (*p* < 0.05).

**Table 3:** The effects of diets combining peanut vine and whole-plant corn silage on slaughter performance and muscle physical properties of Simmental crossbred cattle.

Parameters of Interest ^1^	Treatments			
	WG	LPG	MPG	HPG
Carcass weight, kg	287.75 ± 5.69 ^ab^	285.67 ± 17.61 ^ab^	300.88 ± 4.97 ^a^	268.13 ± 11.71 ^b^
Dressing percentage, %	54.40 ± 1.35	53.84 ± 5.60	53.99 ± 1.27	49.08 ± 2.08
Net meat weight, kg	248.94 ± 6.50 ^ab^	244.86 ± 17.18 ^ab^	261.80 ± 2.49 ^a^	230.65 ± 14.16 ^b^
Carcass meat yield	0.87 ± 0.01	0.86 ± 0.01	0.87 ± 0.01	0.86 ± 0.02
Meat/bone ratio	6.42 ± 0.32	6.01 ± 0.46	6.73 ± 0.48	6.19 ± 0.74
Eye muscle area, cm^2^	90.52 ± 6.50 ^ab^	84.31 ± 9.06 ^b^	106.02 ± 11.23 ^a^	95.95 ± 6.94 ^ab^
Marbling score	1.94 ± 0.42	2.07 ± 0.13	2.67 ± 0.88	2.00 ± 0.58
Cooking yield, %	57.75 ± 6.19	63.00 ± 2.16	61.25 ± 4.92	54.75 ± 5.85
Water holding capacity, %	78.25 ± 2.22	81.00 ± 3.56	81.75 ± 2.22	80.00 ± 3.74
Shear force, kgf	6.00 ± 0.63	5.37 ± 1.16	7.66 ± 3.42	11.31 ± 5.23
pH45min	6.74 ± 0.14	6.71 ± 0.17	6.60 ± 0.14	6.59 ± 0.22
pH48	6.63 ± 0.13	6.67 ± 0.24	6.25 ± 0.44	6.09 ± 0.31

^1^ pH45min, the pH value of the muscle 45 min post-slaughter; pH48, the pH value of the muscle at 48 h post-slaughter. Different letters in the same row indicate significant differences (*p* < 0.05).

**Table 4:** The effects of diets combining peanut vine and whole-plant corn silage on the conventional nutritional composition of the muscle of Simmental crossbred cattle (based on DM).

Parameters of Interest ^1^	Treatments			
	WG	LPG	MPG	HPG
DM (%)	23.92 ± 1.37 ^b^	25.52 ± 1.35 ^ab^	26.57 ± 0.66 ^a^	25.93 ± 0.62 ^ab^
CP (%)	19.29 ± 0.99 ^b^	19.36 ± 0.62 ^b^	21.10 ± 0.74 ^a^	20.31 ± 0.47 ^ab^
EE (%)	3.26 ± 0.54 ^c^	4.94 ± 0.11 ^a^	4.58 ± 0.29 ^ab^	4.16 ± 0.28 ^b^
Ash (%)	1.22 ± 0.06	1.10 ± 0.07	1.25 ± 0.11	1.19 ± 0.03

^1^ EE, ether extract; Ash, crude ash. Different letters in the same row indicate significant differences (*p* < 0.05).

**Table 5:** The effects of diets combining peanut vine and whole-plant corn silage on fatty acid composition of the muscle of Simmental crossbred cattle.

Parameters of Interest ^1^	Treatments			
, mg/g	WG	LPG	MPG	HPG
Myristic acid	2.35 ± 0.64	1.35 ± 0.89	2.01 ± 0.95	1.22 ± 0.86
Palmitic acid	24.15 ± 6.87	23.42 ± 1.80	20.16 ± 5.11	26.72 ± 2.55
Stearic acid	11.47 ± 1.36	16.73 ± 3.38	14.28 ± 2.24	12.99 ± 0.20
Arachidic acid	1.55 ± 0.73	1.39 ± 0.66	1.39 ± 0.66	1.75 ± 0.10
Palmitoleic acid	2.85 ± 0.35	2.83 ± 0.07	3.00 ± 0.47	3.41 ± 0.66
Oleic acid	41.87 ± 0.78	46.28 ± 6.59	46.24 ± 0.37	35.51 ± 5.93
Linoleic acid	5.82 ± 0.05 ^b^	6.69 ± 0.60 ^a^	6.44 ± 0.08 ^ab^	6.71 ± 0.04 ^a^
Arachidonic acid	1.39 ± 0.23	1.60 ± 0.13	1.75 ± 0.18	1.71 ± 0.08
SFA	39.52 ± 9.59	42.89 ± 4.77	37.85 ± 1.26	42.69 ± 3.51
MUFA	44.72 ± 0.43	49.11 ± 6.52	49.24 ± 0.11	39.42 ± 5.97
PUFA	7.21 ± 0.18	8.28 ± 0.47	8.19 ± 0.26	7.92 ± 0.66

^1^ SFA, saturated fatty acids; MUFA, monounsaturated fatty acids; PUFA, polyunsaturated fatty acids. Different letters in the same row indicate significant differences (*p* < 0.05).

**Table 6:** The effects of diets combining peanut vine and whole-plant corn silage on rumen fermentation parameters.

Parameters of Interest ^1^	Treatments			
	WG	LPG	MPG	HPG
pH	6.67 ± 0.09	6.61 ± 0.34	6.82 ± 0.01	6.76 ± 0.21
NH_3_-N, mg/dl	12.08 ± 0.84 ^a^	11.37 ± 1.20 ^a^	7.77 ± 1.10 ^b^	6.95 ± 0.58 ^b^
Acetate, mmol/L	38.31 ± 1.86 ^ab^	38.92 ± 1.46 ^a^	34.68 ± 2.65 ^b^	36.29 ± 2.10 ^ab^
Propionate, mmol/L	18.98 ± 1.22 ^ab^	20.34 ± 2.97 ^a^	16.67 ± 0.71 ^bc^	14.78 ± 0.82 ^c^
Butyrate, mmol/L	6.31 ± 0.59	5.45 ± 0.41	5.91 ± 0.95	6.09 ± 0.93
Valerate, mmol/L	2.71 ± 0.82	2.80 ± 0.24	3.91 ± 1.30	3.42 ± 1.32
Total VFA, mmol/L	66.31 ± 3.36	67.51 ± 4.67	61.17 ± 5.04	60.58 ± 2.87
A/P	2.02 ± 0.04 ^b^	1.94 ± 0.22 ^b^	2.08 ± 0.08 ^b^	2.46 ± 0.14 ^a^

^1^ Total VFA, total volatile fatty acid; A/P, Acetate/Propionate. Different letters in the same row indicate significant differences (*p* < 0.05).

## Data Availability

Data will be made available on request.

## Supplementary Materials

The supplementary materials can be downloaded at: https://www.mdpi.com/article/10.3390/foods12203786/s1, Figure S1: dilution curve of diversity index for rumen microbiota in Simmental crossbreed cattle; Figure S2: (A,B) the alpha diversity of rumen microbiota in Simmental-crossbreed cattle. (A) Shannon index. (B) Chao index. (C) principal coordinate analysis (PCoA). Figure S3: PICRUSt analysis of KEGG function prediction for all treatment groups. Table S1: primer sequences and parameters of genes; Table S2: the effects of diets combining peanut vine and whole-plant corn silage on organ index of Simmental crossbred cattle.
