# Supplementary material for: Effects of Diets Combining Peanut Vine and Whole-Plant Corn Silage on Growth Performance, Meat Quality and Rumen Microbiota of Simmental Crossbred Cattle
Source: Foods. 2023 Oct 15;12(20):3786. doi: 10.3390/foods12203786 (PMC10606686; doi:10.3390/foods12203786)
Supplement: Supplementary file 1 [file foods-12-03786-s001.zip › Supplementary material.pdf]

Table S1. Primer sequences and parameters of genes

Genes	Primer sequence （5'—3'）	Amplification length/bp	Annealing temperature/℃
FAS	F:CGGGATCTGGGTTCACTTGT<br>R:TGCTCACGATACAGGCCTTC	153	60
MDH2	F:TGAAGGATTTGGACCCAGCC<br>R:TGGGGAAATTCCACCTTGGG	110	60
LPL	F:TCACTTCAACCACAGCAGCA<br>R:GATGACGTTGGAGTCCGGTT	127	60
β-actin	F:CACCGCAAATGCTTCTAGGC<br>R:TGTCACCTTCACCGTTCCAG	186	60

Table S2. The effects of diets combining peanut vine and whole-plant corn silage on organ index of Simmental crossbred cattle.

Parameters of interest	Treatments[1]			
	WG	LPG	MPG	HPG
Heart index	0.42±0.05	0.38±0.05	0.40±0.02	0.36±0.05
Liver index	1.18±0.17	1.19±0.13	1.08±0.04	0.98±0.20
Spleen index	0.30±0.10	0.21±0.07	0.18±0.01	0.22±0.05
Lung index	0.51±0.01	0.46±0.06	0.49±0.04	0.49±0.04
Kidney index	0.23±0.01	0.23±0.01	0.22±0.02	0.22±0.04

[1]WG, 45% wheat straw group, n=4; LPG, 25% peanut vine group, n=4; MPG, 45% peanut vine group, n=4; HPG, 65% peanut vine group, n=4.

Different letters in the same row indicate significant differences ($P < 0.05$).

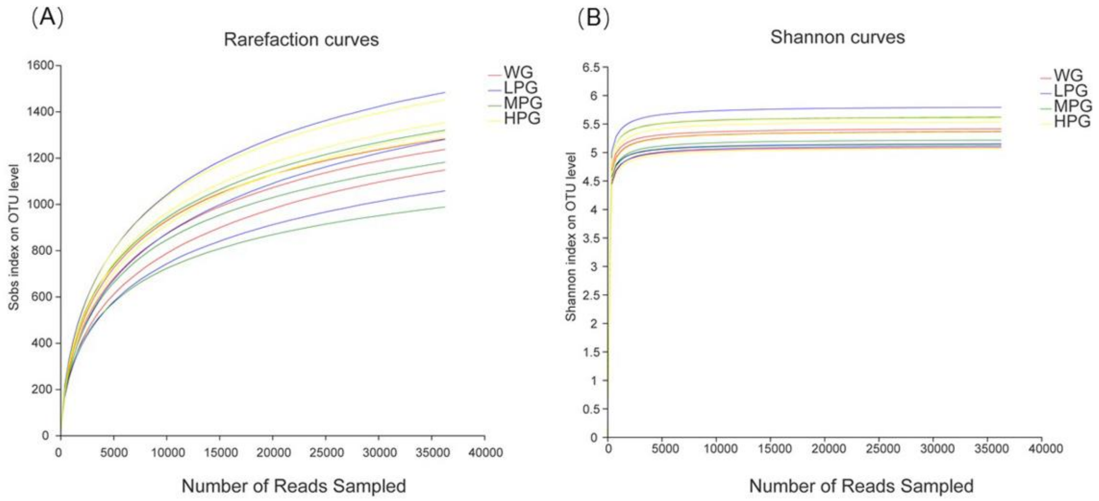

Figure S1. Dilution curve of diversity index for rumen microbiota in Simmental crossbreed cattle. WG, 45% wheat straw group; LPG, 25% peanut vine group; MPG, 45% peanut vine group; HPG, 65% peanut vine group.

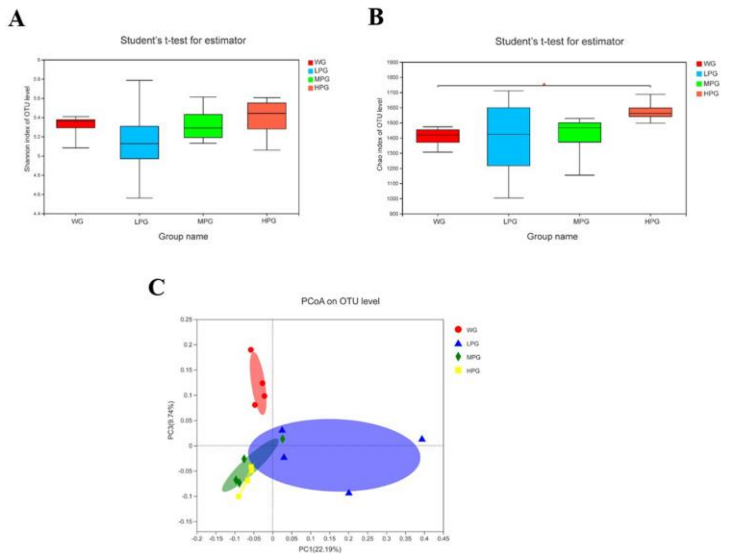

Figure S2. (A-B) The alpha diversity of rumen microbiota in Simmental crossbreed cattle. (A) Shannon index of OTU level. (B) Chao index of OTU level. Error bars represent standard deviations and their lengths are adjusted at 95% confidence interval. (C) Principal coordinate analysis (PCoA) of rumen bacterial community structures of steers in the three groups. PCoA plots based on unweighted UniFrac distance. WG, 45% wheat straw group; LPG, 25% peanut vine group; MPG, 45% peanut vine group; HPG, 65% peanut vine group, n=4. *p < 0.05, **p < 0.01.

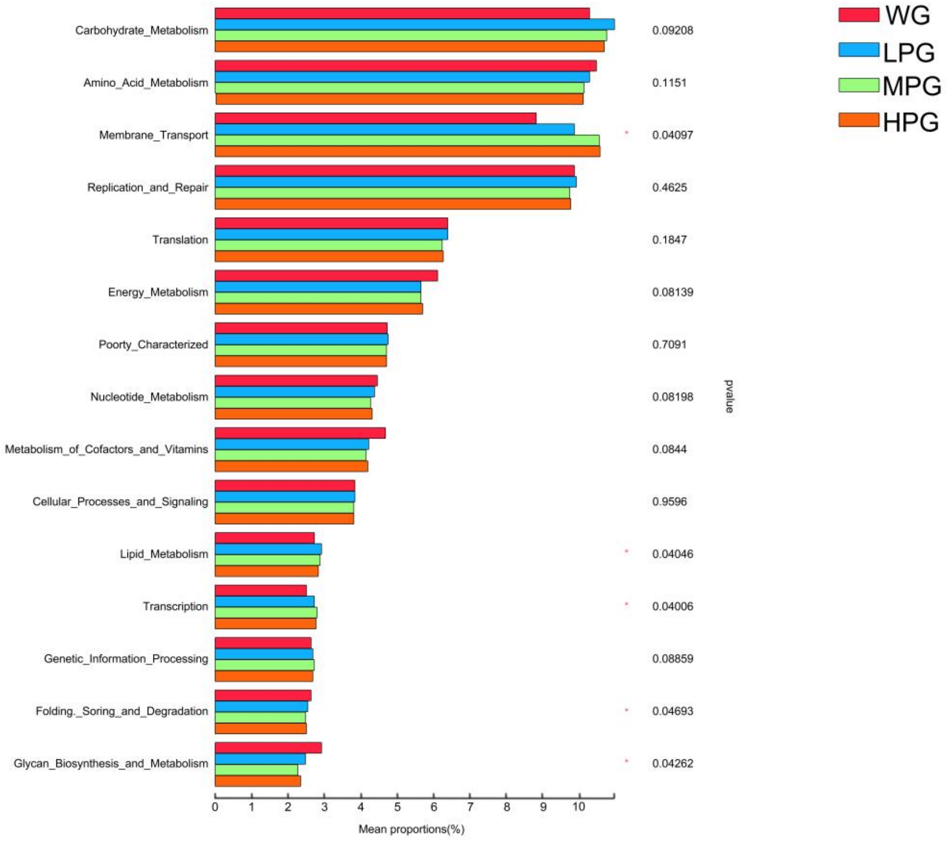

Figure S3. PICRUSt analysis of KEGG function prediction for all treatment groups. WG, 45% wheat straw group; LPG, 25% peanut vine group; MPG, 45% peanut vine group; HPG, 65% peanut vine group, n=4. *p < 0.05, **p < 0.01.
